# Optimal Primary Prophylaxis for Febrile Neutropenia During Neoadjuvant Cisplatin and 5‐Fluorouracil Plus Docetaxel for Esophageal Cancer: A Retrospective Cohort Study

**DOI:** 10.1002/cam4.70889

**Published:** 2025-04-24

**Authors:** Ryosuke Kumanishi, Hiroya Taniguchi, Taiko Nakazawa, Takatsugu Ogata, Yuki Matsubara, Hiroyuki Kodama, Akinobu Nakata, Kazunori Honda, Toshiki Masuishi, Yukiya Narita, Shigenori Kadowaki, Masashi Ando, Masahiro Tajika, Tetsuya Abe, Kei Muro

**Affiliations:** ^1^ Department of Clinical Oncology Aichi Cancer Center Hospital Nagoya Japan; ^2^ Department of Endoscopy Aichi Cancer Center Hospital Nagoya Japan; ^3^ Department of Gastroenterological Surgery Aichi Cancer Center Hospital Nagoya Japan

**Keywords:** 5‐fluorouracil, cisplatin, docetaxel, esophageal cancer, febrile neutropenia, neoadjuvant chemotherapy

## Abstract

**Background:**

Neoadjuvant chemotherapy (NAC) with docetaxel, cisplatin, and 5‐fluorouracil (5‐FU) (DCF) is the standard treatment for locally advanced esophageal cancer (LAEC). DCF is associated with a high risk of febrile neutropenia (FN), but the optimal primary prophylaxis remains unclear. The present study aimed to assess risk factors for FN and efficacy of primary prophylaxis using granulocyte colony‐stimulating factor (G‐CSF) and antibiotics in LAEC patients treated with DCF.

**Methods:**

Patients with LAEC who received DCF as NAC between January 2016 and June 2022 at Aichi Cancer Center Hospital were retrospectively analyzed. DCF consisted of docetaxel 70 mg/m^2^ on day 1, cisplatin 70 mg/m^2^ on day 1, and 5‐FU 750 mg/m^2^ by continuous infusion over 5 days. The patients were divided into Cohort A [no G‐CSF], B1 (G‐CSF after day 6), and B2 (G‐CSFon day 3–4). The efficacy of primary prophylaxis with G‐CSF and antibiotics during the first cycle was evaluated. The potential FN risk factors were evaluated using univariate and multivariate analyses.

**Results:**

Among the 156 patients with esophageal cancer who received DCF as NAC, 41 (26%) patients developed FN during the first cycle. Multivariate analysis revealed that prophylactic antibiotics (17% vs. 40%; adjusted OR, 0.34; 95% confidence interval [CI], 0.16–0.73; *p* = 0.006) and G‐CSF (6% vs. 35%; adjusted OR, 0.14; 95% CI, 0.04–0.47; *p* = 0.002) were associated with lower FN incidence. The grade 3/4 neutropenia rates were 84%, 43%, and 13% in cohorts A, B1, and B2, respectively. FN incidence was 35%, 13%, and 0% in the respective cohorts. Treatment‐related death occurred in 2% of patients, none of whom received G‐CSF prophylaxis.

**Conclusions:**

Prophylactic G‐CSF and antibiotics reduce the risk of FN in patients with LAEC treated with DCF. Early timing of G‐CSF administration showed a potential trend toward reduced FN risk and may offer additional benefits; however, these findings must be validated.

## 1 Introduction

Esophageal cancer is the sixth most frequent cause of cancer‐related deaths worldwide, and its incidence is increasing globally, including in Japan [1, 2]. Although esophagectomy is a curative treatment for patients with locally advanced esophageal cancer (LAEC), the results are unsatisfactory [3]. Several neoadjuvant therapies have been developed to improve treatment outcomes. Neoadjuvant chemoradiotherapy (CRT) is the standard preoperative treatment worldwide [4, 5]. Neoadjuvant chemotherapy (NAC) with cisplatin (CDDP) and 5‐fluorouracil (5‐FU) (CF) has also been a standard treatment and has been commonly used in patients with locally advanced (Stage II or III) esophageal squamous cell carcinoma [6].

Recently, the phase III trial (JCOG1109), which compared the CF arm with the docetaxel, CDDP, and 5‐FU (DCF) arm and the CRT arm as neoadjuvant treatment, demonstrated that the DCF arm had significantly better overall survival than those of the CF arm [7]. Therefore, DCF chemotherapy has become the new standard preoperative treatment for LAEC, particularly for esophageal squamous cell carcinoma (ESCC), which is the primary subtype where DCF is employed. However, severe myelotoxicity, such as neutropenia, resulting in febrile neutropenia (FN) and occasional lethality, is a common side effect that requires caution. In the JCOG 1109 trial, the incidence of FN in the CF, DCF, and CRT arms was 1.0%, 16.3%, and 4.7%, respectively. Despite recommendations for and the extensive use of prophylactic antibiotics, patients in the DCF arm experienced a notably high FN, underscoring a substantial clinical challenge.

Primary prophylaxis with granulocyte colony‐stimulating factor (G‐CSF) is recommended for the prevention of FN in patients who are at risk, such as increased age, medical comorbidities, disease characteristics, and patients receiving high‐risk chemotherapy regimens with FN (FN incidence estimated at greater than 20%) [8, 9]. According to the guidelines, G‐CSF should be administered at least 24 h after completion of chemotherapy. Antibiotic prophylaxis in high‐risk patients is known to decrease the frequency of fever and bacteremia, and quinolone prophylaxis is commonly used in such cases [10]. It has been suggested that delayed administration of G‐CSF as primary prophylaxis may reduce its effectiveness in preventing FN [11]. Reports suggest that early administration of G‐CSF can significantly reduce the FN incidence and is considered a safe approach during DCF therapy [12, 13, 14, 15]. However, G‐CSF administration on day 3 alone may be insufficient to prevent FN, particularly without the concurrent use of prophylactic antibiotics [16]. These studies underscore the potential benefits and limitations of early G‐CSF administration in this context, highlighting the need for further investigation.

Neoadjuvant DCF therapy will be rapidly introduced into clinical practice as a standard of care; however, the optimal prophylaxis for FN remains unclear. Therefore, we conducted this study to assess the effectiveness of primary prophylaxis with G‐CSF and antibiotics to reduce the risk of FN in patients with LAEC treated with DCF, with a particular focus on the impact of G‐CSF use and antibiotics on FN risk, as well as the efficacy of early G‐CSF administration.

## 2 Materials and Methods

### 2.1 Patient Population

This retrospective study evaluated the risk factors for FN in LAEC patients receiving DCF as NAC at Aichi Cancer Center Hospital between January 2016 and June 2022, with a primary focus on the first cycle, and assessed the efficacy and safety of primary prophylaxis with G‐CSF and antibiotics. The eligibility criteria were as follows: 18 years of age or older, Eastern Cooperative Oncology Group performance status (ECOG PS) of 0–1 histologically confirmed resectable esophageal cancer, and no prior history of systemic chemotherapy. Pretreatment staging and resectability were determined at a meeting consisting of thoracic surgeons, radiologists, gastroenterologists, and medical oncologists. The following clinical data were collected: age, sex, ECOG PS, site of primary tumor, UICC TNM classification (8th edition), histological type of tumors, oral intake (adequate or insufficient), dysphagia score, neutrophil/lymphocyte ratio (NLR), creatinine clearance (CCR), total bilirubin (T‐Bil), Glasgow Prognostic Score (GPS), use of primary G‐CSF, and use of prophylactic antibiotics. Inadequate oral intake was defined as requiring daily intravenous fluids or hyperalimentation. The NLR was calculated as the absolute neutrophil count divided by the absolute lymphocyte count. The cutoff values for NLR and T‐Bil were determined according to the median and upper limits of the normal, respectively. Dysphagia score was graded as follows: 0, able to eat a normal diet; 1, able to eat solid food; 2, able to eat semisolid food only; 3, able to swallow liquids only; and 4, complete dysphagia. The GPS was graded as 2 in the presence of both elevated C‐reactive protein (CRP) level (> 1.0 mg/dL) and hypoalbuminemia (< 3.5 g/dL), 1 in the presence of either elevated CRP level or hypoalbuminemia, and 0 in the presence of neither of these. In our study, blood tests were performed at least once during days 6–9 in 95% of the patients (149/156), with no significant differences in the frequency of tests across the three cohorts. All patients provided their written informed consent prior to treatment. This study was approved by the Institutional Review Board of the Aichi Cancer Center Hospital (Approval number: 2021‐0‐251).

### 2.2 Treatment and Assessments

The patients received docetaxel 70 mg/m^2^ on day 1, cisplatin 70 mg/m^2^ on day 1, and 5‐FU 750 mg/m^2^ on days 1–5. The regimen was repeated every 3 weeks. Treatment was continued until completion for three cycles at the physician's discretion because of confirmed disease progression and unacceptable toxicity. Primary prophylaxis with G‐CSF, and oral antibiotics (i.e., levofloxacin) in each cycle was allowed at the physician's discretion. G‐CSF administration was defined as initiation between days 3 and 8. Specifically, filgrastim and pegfilgrastim were administered subcutaneously at 75 or 150 μg and 3.6 mg, respectively. Prophylactic oral antibiotics were administered on days 5–15 in each cycle, based on the physician's judgment. We divided patients without primary G‐CSF administration into Cohort A, those with primary G‐CSF administration on days 6–8 into Cohort B1, and those with primary G‐CSF administration on days 3–4 into Cohort B2. FN was defined as an absolute neutrophil count (ANC) < 1000/μL with an axillary temperature of > 38.0°C. Treatment toxicity was evaluated according to the Common Terminology Criteria for Adverse Events ver. 5.0.

### 2.3 Endpoints and Statistical Analysis

The main objective of this study was to assess the risk factors for FN, estimate FN incidence during the first cycle of treatment, and investigate whether early administration of G‐CSF can reduce the risk of FN. The secondary objectives were to estimate the incidence of grade 3 or 4 neutropenia, depth of the ANC nadir in cycle 1, and incidence of cytotoxic drug dose reduction in cycles 2 to 3.

Clinicopathological factors were compared using Fisher's exact test for categorical variables, the Mann–Whitney *U* test and Kruskal–Wallis test for continuous variables. Furthermore, to understand the risk factors of FN, logistic regression analysis was conducted using background factors as explanatory variables. Given the necessity to consider the use of G‐CSF and antibiotics simultaneously, a multivariate logistic regression analysis was subsequently performed using these two factors. The incidence of FN during the first cycle of treatment, as well as the incidence of grade 3 or 4 neutropenia, was compared between each pair of the three cohorts using Fisher's exact test for two‐group comparisons. The depth of the ANC nadir in cycle 1 and the incidence of cytotoxic drug dose reduction in cycles 2 and 3, expressed as the relative dose intensity, were analyzed using the Kruskal–Wallis test. All statistical analyses were performed using EZR (Saitama Medical Center, Jichi Medical University, Saitama, Japan), a graphical user interface for R (R Foundation for Statistical Computing, Vienna, Austria). All tests were two‐sided, and statistical significance was set at *p* values of < 0.05.

## 3 Results

### 3.1 Patient Characteristics

**FIGURE 1:**
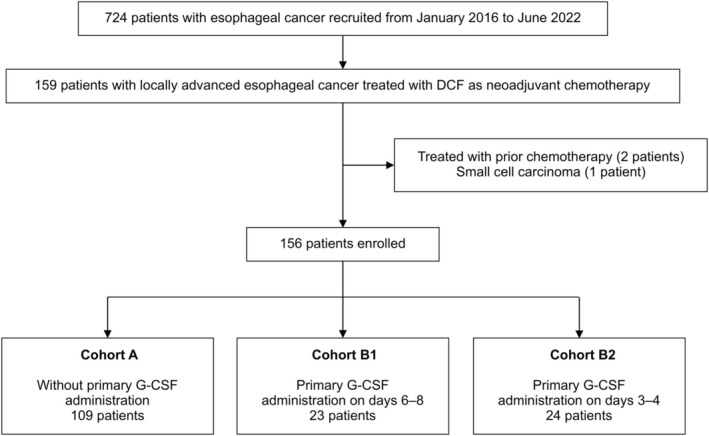
Between January 2016 and June 2022, out of a total of 724 cases of esophageal cancer, 159 patients who underwent neoadjuvant chemotherapy with DCF were enrolled. Of these, 3 patients were excluded due to not meeting the inclusion criteria, resulting in a final cohort of 156 patients. These patients were then categorized into different cohorts based on the use of G‐CSF.

Among the 724 esophageal cancer patients, 156 patients who started DCF as NAC were included. The last follow‐up time was August 2022. The study cohort (Table 1) included 126 men and 30 women with a median age of 67 years (range, 35–79), and the ECOG PS score was 0 in 99 patients and 1 in 57 patients. Prophylactic antibiotics were used in 93 patients, and G‐CSF was used as primary prophylaxis in 47 patients. No significant differences in the baseline characteristics between the groups were noted, except for the location of the primary tumor, creatinine clearance, and the presence of initial dose reduction (Table S1). Filgrastim administration was initiated between days 3–8, and the course of treatment lasted from 4 to 9 days following the onset, and peg filgrastim was given once per cycle on any single day between days 3–8. Cohort A comprised 109 cases, Cohort B1 comprised 23 cases, and Cohort B2 comprised 24 cases (Figure 1).

**TABLE 1:** Baseline patient characteristics.

*n* = 156	All
Median age, years (range)	67 (35–79)
Sex, *n* (%)	
Male	126 (81)
Female	30 (19)
ECOG PS, *n* (%)	
0	99 (63)
1	57 (37)
Site of primary tumor, *n* (%)	
Cervical/upper thoracic	44 (28)
Middle thoracic	75 (48)
Lower thoracic/abdominal	37 (24)
Clinical Stage (UICC 8th), *n* (%)	
I–II	7 (5)
III	86 (55)
IVA	14 (9)
IVB	49 (31)
Clinical T stage, *n* (%)	
T1–T2	14 (9)
T3	123 (79)
T4	19 (12)
Histological type, *n* (%)	
Squamous carcinoma	151 (97)
Adenocarcinoma	5 (3)
Dysphagia score, *n* (%)	
0	72 (46)
1–3	68 (44)
4	16 (10)
Creatinine clearance, *n* (%)	
< 50	17 (11)
≥ 50	139 (89)
Glasgow Prognostic Score^a^, *n* (%)	
0–1	128 (82)
2	27 (17)
Missing	1 (1)
Initial dose reduction, *n* (%)	
Yes	21 (13)
No	135 (87)
Primary G‐CSF use, *n* (%)	
Yes	47 (30)
No	109 (70)
Prophylactic Antibiotics^b^, *n* (%)	
Yes	93 (60)
No	63 (40)

Abbreviations: ECOG PS, Eastern Cooperative Oncology Group Performance Status; UICC, Union for International Cancer Control.

^a^ Score 0: CRP ≤ 1.0 and Alb ≥ 3.5, Score 1: CRP > 1.0 or Alb < 3.5, Score 2: CRP > 1.0 and Alb < 3.5.

^b^ Prophylactic quinolone antibiotics were used on days 5–14.

### 3.2 Treatment Administered

Twenty‐two patients discontinued treatment after the first cycle, 16 discontinued after the second cycle, and 118 patients completed three cycles of DCF therapy. Finally, 116 of the 156 patients underwent esophagectomy. The surgery completion rates were 72% (79/109), 83% (19/23), and 75% (18/24) in Cohort B2, with no significant differences observed among the groups (Cohort A vs. B1, *p* = 0.43; Cohort A vs. B2, *p* = 1.00; Cohort B1 vs. B2, *p* = 0.72). The reasons for not completing three cycles of DCF included 21 cases of tumor progression, four adverse events (AEs), and three treatment‐related deaths in Cohort A; two cases of tumor progression and one AE in Cohort B1; and six cases of tumor progression and one AE in Cohort B2. The chemotherapy completion rates for three cycles of DCF therapy were 74% (81/109) in Cohort A, 87% (20/23) in Cohort B1, and 71% (17/24) in Cohort B2, with no significant differences observed among the groups (Cohort A vs. B1, *p* = 0.28; Cohort A vs. B2, *p* = 0.80; Cohort B1 vs. B2, *p* = 0.29). The median relative dose intensities of the drugs over all cycles in the groups are shown in Table 2. There were no statistically significant differences in the total or relative dose intensities among the three groups.

**TABLE 2:** Relative dose intensity (Cycles 1–3).

5‐FU (%)			
*n* = 156	Cohort A	Cohort B1	Cohort B2
	*n* = 109	*n* = 23	*n* = 24
Median	86	85	89
Range	56–100	70–100	62–102
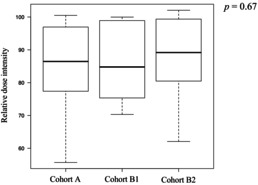			

Cisplatin (%)			
*n* = 156	Cohort A	Cohort B1	Cohort B2
	*n* = 109	*n* = 23	*n* = 24
Median	85	84	86
Range	49–100	45–100	51–102
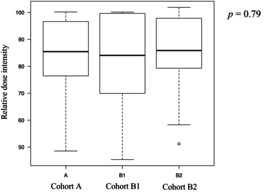			

Docetaxel (%)			
*n* = 156	Cohort A	Cohort B1	Cohort B2
	*n* = 109	*n* = 23	*n* = 24
Median	86	91	89
Range	54–100	70–100	66–102
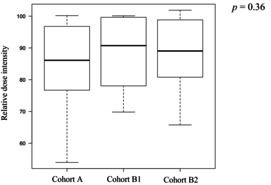			

### 3.3 Incidence of FN With or Without G‐CSF Use

**FIGURE 2:**
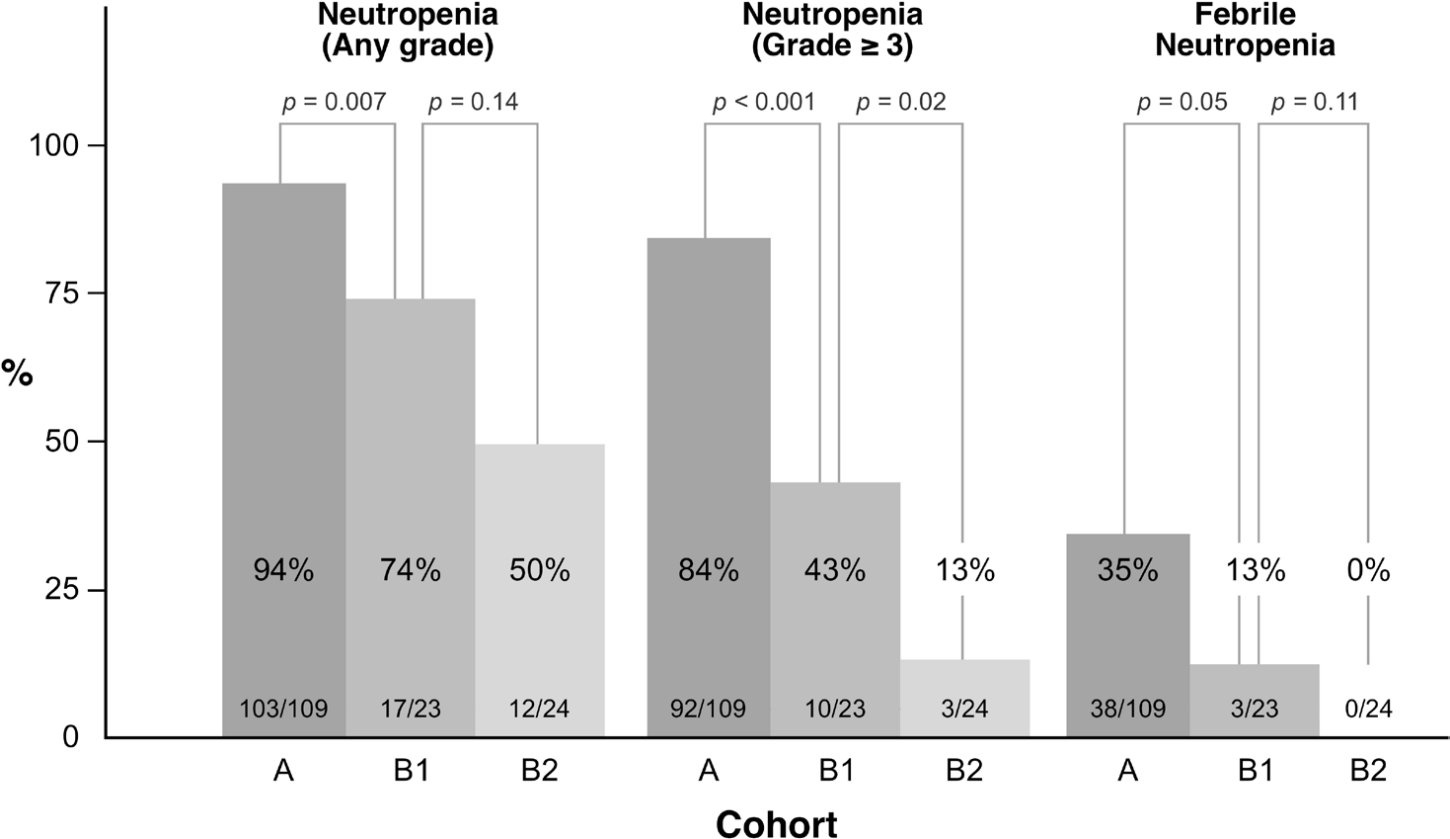
It was demonstrated that the risk for all categories of neutropenia (any grade), severe neutropenia (grade 3 or above), and febrile neutropenia was lowest in the Cohort B2 group.

A total of 41 patients (26%) developed FN on the median day of 8 (range, 5–12) of the first cycle, of which 40 developed grade 3 and one developed grade 4. The incidence rates of FN were 35%, 13%, and 0% in Cohorts A, B1, and B2, respectively (Cohort A vs. B1, *p* = 0.05; Cohort A vs. B2, *p* < 0.001) (Figure 2), and the FN incidence tended to be lower in Cohort B2 than in those with Cohort B1 (0% vs. 13%, *p* = 0.11). The median durations of hospitalization were 12, 10, and 11 days in Cohorts A, B1, and B2, respectively (*p* = 0.09), with no significant differences among the cohorts. The incidence rates of grade 3 or 4 neutropenia were 84%, 43%, and 13% in Cohorts A, B1, and B2, respectively (Cohort A vs. B1, *p* < 0.001; Cohort A vs. B2, *p* < 0.001; Cohort B1 vs. B2, *p* = 0.02).

Furthermore, we conducted a subgroup analysis within Cohorts B1 and B2, categorizing patients into the filgrastim group and pegfilgrastim group. In Cohort B1, the incidence of grade 3 or 4 neutropenia was significantly higher in the filgrastim group than in the pegfilgrastim group (86% vs. 25%, *p* = 0.02), whereas that of FN was 0% in the filgrastim group and 19% in the pegfilgrastim group (*p* = 0.53). In Cohort B2, the incidence of grade 3 or 4 neutropenia was 67% in the filgrastim group compared with 5% in the pegfilgrastim group (*p* = 0.03), with no cases of FN observed in either group (0% vs. 0%, *p* = 1.00).

Additionally, the cumulative incidence of FN across cycles 1–3 was 39% (42/109) in Cohort A, 13% (3/23) in Cohort B1, and 0% (0/24) in Cohort B2. The differences between the early and late administration groups were not statistically significant (Cohort A vs. B1, *p* = 0.03; Cohort A vs. B2, *p* < 0.001; Cohort B1 vs. B2, *p* = 0.11).

### 3.4 Risk Factors Analysis for FN Using Multivariate Analysis

Next, we examined potential additional risk factors for FN in the first cycle in 156 patients. Univariate analysis identified the use of primary G‐CSF and prophylactic antibiotics as significant factors associated with reducing the risk of FN, with odds ratios of 0.13 (*p* < 0.001) and 0.32 (*p* = 0.002), respectively. Other factors, such as age and ECOG PS, were not significant risk factors for FN. Moreover, multivariate analysis showed that prophylactic antibiotic use [17% vs. 40%; adjusted odds ratio (OR), 0.34; 95% confidence interval (CI), 0.16–0.73; *p* = 0.006] and G‐CSF (6% vs. 35%; adjusted OR, 0.14; 95% CI, 0.04–0.47; *p* = 0.002) were independently associated with a lower FN incidence (Table 3).

**TABLE 3:** Uni‐ and multivariate analyses of febrile neutropenia frequency.

Characteristics	Febrile neutropenia frequency	Univariate analysis			Multivariate analysis		
		Odds ratio	95% CI	*p*	Odds ratio	95% CI	*p*
Age (≥ 70 vs. < 70)	30% (17/57) vs. 24% (24/99)	1.33	0.64–2.76	0.45			
ECOG PS (1 vs. 0)	25% (14/57) vs. 27% (27/99)	0.87	0.41–1.83	0.71			
Location (Ce/Ut vs. Mt/Ae)	21% (9/44) vs. 29% (32/112)	0.64	0.28–1.49	0.30			
Clinical T stage (T4 vs. T1–3)	26% (5/19) vs. 26% (36/137)	1.00	0.34–2.98	1.00			
Dysphagia Score (4 vs. 0–3)	31% (5/16) vs. 26% (36/140)	1.31	0.43–4.04	0.63			
Creatinine clearance (< 50 vs. ≥ 50)	18% (3/17) vs. 27% (38/139)	0.57	0.16–2.10	0.40			
Glasgow Prognostic Score (2 vs. 0–1)	26% (7/27) vs. 27% (34/128)	0.97	0.38–2.49	0.95			
Initial dose reduction (Yes vs. No)	19% (4/21) vs. 27% (37/135)	0.62	0.20–1.97	0.42			
Primary G‐CSF use (Yes vs. No)	6% (3/47) vs. 35% (38/109)	0.13	0.04–0.44	< 0.001	0.14	0.04–0.47	0.002
Prophylactic Antibiotics (Yes vs. No)	17% (16/93) vs. 40% (25/63)	0.32	0.15–0.66	0.002	0.33	0.15–0.72	0.005

Abbreviations: Ae, abdominal esophagus; Ce, cervical esophagus; CI, confidence interval; ECOG, PS Eastern Cooperative Oncology Group performance status; Lt, lower thoracic esophagus; Mt, middle thoracic esophagus; Ut, upper thoracic esophagus.

### 3.5 Safety

The overall AEs, excluding neutropenia and FN, are summarized in Table 4. The most common hematological toxicities were anemia (92%), hyponatremia (89%), and thrombocytopenia (64%). The most common nonhematological toxicities were anorexia (78%), fatigue (62%), and nausea (57%). No statistically significant differences were found among the three cohorts in terms of hematological or nonhematological toxicities. Two patients in Cohort A developed a treatment‐related esophageal fistula. There were three cases of treatment‐related death, all of which occurred in Cohort A. The causes were sepsis resulting from fungal pneumonia in one patient, primary hemorrhage associated with FN in another, and tracheal infiltration leading to perforation in the third. G‐CSF‐related bone pain or back pain was observed in one patient in Cohort B2. This case was mild and rapidly resolved. No serious AEs related to the G‐CSF administration were observed.

**TABLE 4:** Adverse events.

	Cohort A (*n* = 109)		Cohort B1 (*n* = 23)		Cohort B2 (*n* = 24)		*p*	*p*	All (*n* = 156)	
	Any grade	Grade ≥ 3	Any grade	Grade ≥ 3	Any grade	Grade ≥ 3	A vs. B1	A vs. B2	Any grade	Grade ≥ 3
	*n* (%)	*n* (%)	*n* (%)	*n* (%)	*n* (%)	*n* (%)	Grade ≥ 3	Grade ≥ 3	*n* (%)	*n* (%)
Hematological										
Neutropenia	103 (94)	92 (84)	17 (74)	10 (43)	12 (50)	3 (13)	< 0.001	< 0.001	132 (85)	105 (67)
Anemia	102 (94)	4 (4)	21 (91)	0 (0)	21 (88)	0 (0)	1.00	1.00	144 (92)	4 (3)
Thrombocytopenia	61 (56)	5 (5)	17 (74)	1 (4)	22 (92)	2 (8)	1.00	0.61	100 (64)	8 (5)
Febrile neutropenia	38 (35)	38 (35)	3 (13)	3 (13)	0 (0)	0 (0)	0.05	< 0.001	41 (26)	41 (26)
AST increased	47 (43)	3 (3)	7 (30)	0 (0)	4 (17)	0 (0)	1.00	1.00	58 (37)	3 (2)
ALT increased	47 (43)	2 (2)	6 (26)	0 (0)	5 (21)	0 (0)	1.00	1.00	58 (37)	2 (1)
LDH increased	17 (16)	0 (0)	6 (26)	0 (0)	5 (21)	0 (0)	1.00	1.00	28 (18)	0 (0)
Creatinine increased	21 (19)	0 (0)	5 (22)	0 (0)	4 (17)	0 (0)	1.00	1.00	30 (19)	0 (0)
Hyponatremia	102 (94)	8 (7)	19 (83)	1 (4)	18 (75)	2 (8)	1.00	1.00	139 (89)	11 (7)
Nonhematological										
Anorexia	92 (84)	12 (11)	13 (57)	0 (0)	17 (71)	1 (4)	0.21	0.69	122 (78)	13 (8)
Fatigue	71 (65)	3 (3)	13 (57)	0 (0)	13 (54)	0 (0)	1.00	1.00	97 (62)	3 (2)
Nausea	71 (65)	3 (3)	8 (35)	0 (0)	10 (42)	0 (0)	1.00	1.00	89 (57)	3 (2)
Vomiting	11 (10)	1 (1)	0 (0)	0 (0)	1 (4)	0 (0)	1.00	1.00	12 (8)	1 (1)
Diarrhea	35 (32)	6 (6)	5 (22)	0 (0)	7 (29)	1 (4)	0.59	1.00	47 (30)	7 (4)
Oral mucositis	18 (17)	1 (1)	5 (22)	0 (0)	4 (17)	0 (0)	1.00	1.00	27 (17)	1 (1)
Esophageal fistula	2 (2)	2 (2)	0 (0)	0 (0)	0 (0)	0 (0)	1.00	1.00	2 (1)	2 (1)
Bone pain or back pain	0 (0)	0 (0)	0 (0)	0 (0)	1 (4)	0 (0)	1.00	1.00	1 (1)	0 (0)
Arthritis	1 (1)	0 (0)	0 (0)	0 (0)	1 (4)	0 (0)	1.00	1.00	2 (1)	0 (0)
Pneumonia	1 (1)	1 (1)	0 (0)	0 (0)	0 (0)	0 (0)	1.00	1.00	1 (1)	1 (1)

Abbreviations: ALT, alanine aminotransferase; AST, aspartate aminotransferase; LDH, lactate dehydrogenase.

## 4 Discussion

In the present retrospective study, the prophylactic use of antibiotics and G‐CSF was observed to be associated with a lower incidence of FN in patients with ESCC receiving neoadjuvant DCF therapy. Furthermore, these administrations were identified as independent risk factors for FN. Additionally, the FN risk was lower in patients who received G‐CSF early than in those who received it after day 5, but the difference was not significant.

The present study demonstrated the efficacy of primary prophylaxis with G‐CSF. Although meta‐analyses have demonstrated the mortality‐reduction effects of prophylactic G‐CSF administration [17], the guidelines do not recommend a detailed dosing plan for G‐CSF during the course of continuous chemotherapy for several days [8, 9]. According to these guidelines, G‐CSF should be administered 1–3 days after completion of chemotherapy. There is a lack of data on the concomitant use of G‐CSF and chemotherapy, and the optimal dosing schedule of G‐CSF remains unclear, especially in regimens involving continuous infusions of 5‐fluorouracil, such as the DCF regimen. In a phase II study to assess the efficacy and safety of early dosing of pegfilgrastim for patients with advanced ESCC, of the 19 patients who received two cycles of DCF, no patient experienced FN and no serious AEs considered relevant to pegfilgrastim were observed [12]. Previous retrospective studies have suggested that pegfilgrastim administration on day 3 in the context of NAC DCF therapy for LAEC may not be sufficient to prevent FN [16].

In the present retrospective study, the frequency of FN in patients with primary G‐CSF administration on days 3 or 4 tended to be lower than that in those without primary G‐CSF administration and those with primary G‐CSF administration after day 5. Previous studies investigating the timing and efficacy of pegfilgrastim administration in patients undergoing DCF therapy have defined early administration as that performed on days 3–5 postchemotherapy [15]. Our study also found a trend toward lower rates of FN and grade 3–4 neutropenia in the early administration group, but this result might not be specifically attributable to the timing of administration on days 3–4. Considering the rapid onset of neutropenia after DCF therapy, further studies are warranted to clarify the optimal timing of G‐CSF administration. However, data on this approach remain limited, so further prospective studies are needed to clearly demonstrate the benefits of G‐CSF administration versus later or standard administration.

Previous studies have suggested that prophylactic pegfilgrastim administration may reduce the risk of FN compared with filgrastim [18, 19]. Similarly, our study indicates that pegfilgrastim may lower the incidence of grade 3 or higher neutropenia compared with filgrastim. Therefore, although pegfilgrastim might be the better alternative compared with filgrastim, it is important to note that the sample size of our study was small, and we found no significant difference in FN incidence. Caution should be exercised when interpreting these results, and further research with larger cohorts should be conducted.

In this study, 60% of patients received antibiotic prophylaxis. The administration of prophylactic antibiotics during the neutropenic phase following chemotherapy has been shown to significantly decrease the occurrence of fever and sepsis [20, 21, 22]. Interestingly, the multivariate analysis in our study identified antibiotic use as an independent risk factor for FN, separate from G‐CSF use. Unlike our study, previous studies [12, 15] and the JCOG1109 trial [7] reported the use of antibiotics in almost all cases, leaving the significance of antibiotic use under G‐CSF administration unclear. Our findings suggest the importance of continuing antibiotic prophylaxis even in patients who receive G‐CSF via DCF therapy. However, the possibility that antibiotics use may reduce the efficacy of immune checkpoint inhibitors should be considered [23, 24]. As immune checkpoint inhibitors become more commonly coadministered in the future, the decision to use antibiotics will require careful reevaluation.

Multivariate analysis identified no significant risk factors related to FN in the present study, except for the non‐use of prophylactic antibiotics and G‐CSF. While guidelines indicate advanced age, poor PS, poor renal/liver function, and non‐use of G‐CSF or prophylactic antibiotics as risk factors for FN [8, 9, 25], these were not statistically significant in our cohort. Although the limited number of patients may have influenced the results, this suggests that non‐use of prophylactic antibiotics and G‐CSF are the most important factors among the identified risk factors.

AEs reported in patients receiving G‐CSF were bone pain and arthritis, although the incidence of these AEs was lower than previously reported [26, 27, 28]. The reason behind this disparity remains unclear, but one possible explanation could be that bone pain and arthritis are well‐recognized AEs associated with the use of G‐CSF, and as a result, it might be less frequently reported in the retrospective analysis. Considering that there was no increase in other AEs in this study, the prophylactic administration of G‐CSF is not expected to increase the risk to patients.

The present study had several limitations. First, it was a retrospective, nonrandomized analysis with a small number of patients. Second, there are no rules regarding the prophylactic use of antibiotics or G‐CSF. In previous studies, almost all patients received prophylactic antibiotics; however, in the present study, the prophylactic use of antibiotics and G‐CSF was the physician's choice. Third, the applicability of the NAC DCF regimen may vary depending on the predominant histology of esophageal cancer in different regions. Although this regimen is standard for ESCC in Japan, its generalizability to regions where adenocarcinoma is more prevalent (such as in Western countries) may be limited. Additionally, because blood tests were not conducted daily before and after the nadir of neutrophil counts, the incidence of FN may have been underestimated. Even a 1‐day variation in test timing can significantly impact neutrophil count measurements. Given the retrospective nature of this study, this potential limitation should be considered when interpreting FN incidence rates.

In conclusion, the present study suggests that the prophylactic use of G‐CSF and antibiotics may be associated with a reduced risk of FN in patients with LAEC treated with DCF. Although the findings indicate that early administration of primary G‐CSF during DCF therapy may reduce the risk of severe neutropenia and FN, the results should be interpreted with caution due to the study's limitations. Additional studies with larger cohorts are warranted to confirm these observations with more robust evidence.

## Author Contributions

**Ryosuke Kumanishi:** writing – original draft (lead). **Hiroya Taniguchi:** writing – review and editing (equal). **Taiko Nakazawa:** writing – review and editing (equal). **Takatsugu Ogata:** writing – review and editing (equal). **Yuki Matsubara:** writing – review and editing (equal). **Hiroyuki Kodama:** writing – review and editing (equal). **Akinobu Nakata:** writing – review and editing (equal). **Kazunori Honda:** writing – review and editing (equal). **Toshiki Masuishi:** writing – review and editing (equal). **Yukiya Narita:** writing – review and editing (equal). **Shigenori Kadowaki:** writing – review and editing (equal). **Masashi Ando:** writing – review and editing (equal). **Masahiro Tajika:** writing – review and editing (equal). **Tetsuya Abe:** writing – review and editing (equal). **Kei Muro:** writing – review and editing (equal).

## Ethics Statement

All procedures performed in this study involving human participants were conducted in accordance with the ethical standards of the institutional and national research committee and the 1964 Helsinki declaration and its later amendments or comparable ethical standards. This study was approved by the Institutional Review Board of the Aichi Cancer Center Hospital.

## Consent

All patients provided written informed consent prior to treatment.

## Conflicts of Interest

Ryosuke Kumanishi has received honoraria from Ono Pharmaceutical Co. Ltd., Taiho Pharmaceutical Co. Ltd., and Merck Biopharma. Hiroya Taniguchi has received honoraria from Ono Pharmaceutical Co. Ltd., Taiho Pharmaceutical Co. Ltd., Eli Lilly Japan K.K., Chugai Pharmaceutical Co. Ltd., Bristol–Myers Squibb, Takeda, Merck Biopharma, Amgen, and Roche Diagnostics, and research funding from Takeda and Daiichi Sankyo. Yuki Matsubara has received honoraria from Taiho Pharmaceutical Co. Ltd., Takeda, Eli Lilly Japan K.K., and Bristol–Myers Squibb. Akinobu Nakata has received honoraria from Novartis Pharmaceutical Co. Kazunori Honda has received research funding from Pfizer Japan Inc. outside the context of the submitted work. Toshiki Masuishi has received honoraria from Bayer Yakuhin, Bristol–Myers Squibb, Chugai Pharmaceutical Co. Ltd., Daiichi Sankyo, Eli Lilly, Merck Serono, Ono Pharmaceutical, Sanofi, Taiho Pharmaceutical Co. Ltd., Takeda, Yakult Honsha, MSD, and Nippon Kayaku, and research funding from Amgen, Boehringer Ingelheim, CMIC, Daiichi Sankyo, Lilly Japan, MSD, Novartis, Ono Pharmaceutical, Pfizer, and Syneos Health. Yukiya Narita has received grants and personal fees from Ono Pharmaceutical, Bristol–Myers Squibb, AstraZeneca, and Daiichi Sankyo, and honoraria from Yakult Honsha, Taiho Pharmaceutical Co. Ltd., Eli Lilly, Daiichi Sankyo, Ono Pharmaceutical, and Bristol–Myers Squibb, and participation on an advisory board from Daiichi Sankyo. Shigenori Kadowaki has received honoraria from Bristol–Myers Squibb, Ono Pharmaceutical, Bayer, Merck Biopharma, Taiho Pharmaceutical Co. Ltd., Eisai, Daiichi Sankyo, MSD, Chugai Pharmaceutical Co. Ltd., and Otsuka Pharmaceutical, and research funding from Bristol–Myers Squibb, Ono Pharmaceutical, Bayer, Daiichi Sankyo, MSD, Chugai Pharmaceutical Co. Ltd., Janssen, Nobelpharma, AstraZeneca, and Eli Lilly. Masashi Ando has received honoraria from Eisai, Ono Pharmaceutical, Chugai Pharmaceutical Co. Ltd., and Taiho Pharmaceutical Co. Ltd. Masahiro Tajika has received honoraria from EA Pharma Co. Ltd. and Otsuka Pharmaceutical. Kei Muro has received research funding from Amgen, Ono Pharmaceutical, Astellas, Sanofi, Taiho Pharmaceutical Co. Ltd., PRA Health Sciences, PAREXEL International, Novartis, Chugai Pharmaceutical Co. Ltd., MSD, and consulting fees from Amgen, AstraZeneca, Ono Pharmaceutical, Astellas, Chugai Pharmaceutical Co. Ltd., and honoraria from Ono Pharmaceutical, Taiho Pharmaceutical Co. Ltd., Bristol–Myers Squibb, Eli Lilly, MSD, Takeda, Daiichi Sankyo, and advisory board from Astellas, Amgen, AstraZeneca, and Takeda. The other authors declare no conflicts of interest.

## Supporting information


Table S1.

## Data Availability

The data that support the findings of this study are available from the corresponding author upon reasonable request.
