# Supplementary material for: Optimal Primary Prophylaxis for Febrile Neutropenia During Neoadjuvant Cisplatin and 5‐Fluorouracil Plus Docetaxel for Esophageal Cancer: A Retrospective Cohort Study
Source: Cancer Med. 2025 Apr 24;14(9):e70889. doi: 10.1002/cam4.70889 (PMC12021990; doi:10.1002/cam4.70889)
Supplement: Supplementary file 1 — Table S1. [file CAM4-14-e70889-s001.docx]

# **Supplementary Table 1: Baseline characteristics by cohort**

| ***n* = 156** | **Cohort A (*n* = 109)** | **Cohort B1 (*n* = 23)** | **Cohort B2 (*n* = 24)** | ***p*** |
| --- | --- | --- | --- | --- |
| Median age, years | 67 (42–79) | 65 (35–79) | 68 (47–79) | 0.21 |
| (range) |  |  |  |  |
| Sex, *n* (%) |  |  |  | 0.29 |
| Male | 91 (83) | 18 (78) | 17 (71) |  |
| Female | 18 (17) | 5 (22) | 7 (29) |  |
| ECOG PS, *n* (%) |  |  |  | 0.49 |
| 0 | 66 (61) | 17 (74) | 16 (67) |  |
| 1 | 43 (39) | 6 (26) | 8 (33) |  |
| Site of primary tumor, *n* (%) |  |  |  | 0.003 |
| Ce–Ut | 25 (23) | 13 (57) | 6 (25) |  |
| Mt | 54 (50) | 6 (26) | 15 (63) |  |
| Lt–Ae | 30 (28) | 4 (17) | 3 (13) |  |
| Clinical stage (UICC 8th), *n* (%) |  |  |  | 0.14 |
| I–II | 4 (4) | 2 (9) | 1 (4) |  |
| III | 67 (61) | 10 (43) | 9 (38) |  |
| IVA | 10 (9) | 1 (4) | 3 (13) |  |
| IVB | 28 (26) | 10 (43) | 11 (46) |  |
| Clinical T stage, *n* (%) |  |  |  | 0.35 |
| T1–T2 | 7 (6) | 3 (13) | 4 (17) |  |
| T3 | 89 (82) | 16 (70) | 18 (75) |  |
| T4 | 13 (12) | 4 (17) | 2 (8) |  |
| Histological type, *n* (%) |  |  |  | 0.48 |
| Squamous carcinoma | 106 (97) | 22 (96) | 23 (96) |  |
| Adenocarcinoma | 3 (3) | 1 (4) | 1 (4) |  |
| Oral Intake, *n* (%) |  |  |  | 0.42 |
| Sufficient | 98 (90) | 22 (96) | 20 (83) |  |
| Insufficient | 11 (10) | 1 (4) | 4 (17) |  |
| Dysphagia Score, *n* (%) |  |  |  | 0.09 |
| 0 | 39 (36) | 16 (70) | 17 (71) |  |
| 1–3 | 59 (54) | 6 (26) | 3 (13) |  |
| 4 | 11 (10) | 1 (4) | 4 (17) |  |
| Neutrophil lymphocyte ratio, *n* (%) |  |  |  | 0.06 |
| ≥2.5 | 72 (66) | 12 (52) | 21 (88) |  |
| <2.5 | 36 (33) | 11 (48) | 3 (13) |  |
| Missing | 1 (1) | 0 (0) | 0 (0) |  |
| Creatinine clearance, *n* (%) |  |  |  | 0.02 |
| <50 | 7 (6) | 5 (22) | 5 (21) |  |
| ≥50 | 102 (94) | 18 (78) | 19 (79) |  |
| Total bilirubin, *n* (%) |  |  |  | 0.43 |
| <1.5 mg/dL | 107 (98) | 22 (96) | 24 (100) |  |
| ≥1.5 mg/dL | 2 (2) | 1 (4) | 0 (0) |  |
| Glasgow Prognostic Score, *n* (%) |  |  |  | 0.80 |
| 0–1 | 86 (79) | 21 (91) | 21 (88) |  |
| 2 | 22 (20) | 2 (9) | 3 (13) |  |
| Missing | 1 (1) | 0 (0) | 0 (0) |  |
| Initial dose reduction, *n* (%) |  |  |  | 0.04 |
| Yes | 11 (10) | 7 (30) | 3 (13) |  |
| No | 98 (90) | 16 (70) | 21 (88) |  |
| Primary G-CSF, *n* (%) |  |  |  |  |
| Filgrastim | - | 7 (30) | 3 (13) |  |
| Pegfilgrastim | - | 16 (70) | 21 (88) |  |
| Prophylactic Antibiotics, *n* (%) |  |  |  | 0.11 |
| Yes | 61 (56) | 13 (57) | 19 (79) |  |
| No | 48 (44) | 10 (43) | 5 (21) |  |
